# DDIT4L regulates mitochondrial and innate immune activities in early life

**DOI:** 10.1172/jci.insight.172312

**Published:** 2024-02-06

**Authors:** Christina Michalski, Claire Cheung, Ju Hee Oh, Emma Ackermann, Constantin R. Popescu, Anne-Sophie Archambault, Martin A. Prusinkiewicz, Rachel Da Silva, Abdelilah Majdoubi, Marina Viñeta Paramo, Rui Yang Xu, Frederic Reicherz, Annette E. Patterson, Liam Golding, Ashish A. Sharma, Chinten J. Lim, Paul C. Orban, Ramon I. Klein Geltink, Pascal M. Lavoie

**Affiliations:** 1 British Columbia Children’s Hospital Research Institute, Vancouver, British Columbia, Canada.; 2 Department of Pediatrics and; 3 Department of Pathology and Laboratory Medicine, University of British Columbia, Vancouver, British Columbia, Canada.; 4 Department of Pediatrics, Université Laval, Quebec, Quebec, Canada.; 5 Women+ and Children′s Health, Department of Obstetrics and Gynecology, University of British Columbia, Vancouver, British Columbia, Canada.; 6 Department of Pathology and Laboratory Medicine, Emory University, Atlanta, Georgia, USA.; 7 Department of Surgery, University of British Columbia, Vancouver, British Columbia, Canada.

**Keywords:** Immunology, Bioenergetics, Cellular immune response, Monocytes

## Abstract

Pattern recognition receptor responses are profoundly attenuated before the third trimester of gestation in the relatively low-oxygen human fetal environment. However, the mechanisms regulating these responses are uncharacterized. Herein, genome-wide transcription and functional metabolic experiments in primary neonatal monocytes linked the negative mTOR regulator DDIT4L to metabolic stress, cellular bioenergetics, and innate immune activity. Using genetically engineered monocytic U937 cells, we confirmed that DDIT4L overexpression altered mitochondrial dynamics, suppressing their activity, and blunted LPS-induced cytokine responses. We also showed that monocyte mitochondrial function is more restrictive in earlier gestation, resembling the phenotype of DDIT4L-overexpressing U937 cells. Gene expression analyses in neonatal granulocytes and lung macrophages in preterm infants confirmed upregulation of the *DDIT4L* gene in the early postnatal period and also suggested a potential protective role against inflammation-associated chronic neonatal lung disease. Taken together, these data show that DDIT4L regulates mitochondrial activity and provide what we believe to be the first direct evidence for its potential role supressing innate immune activity in myeloid cells during development.

## Abstract 2

The negative mTOR regulator DDIT4L has a developmental role in human neonatal myeloid cell by limiting cytokine production and reconfiguring mitochondrial dynamics.

## Introduction

The fetal immune system has a remarkable ability to tolerate maternal semiallogeneic antigens, in part through lowered innate immune activity, the presence of prominent antiinflammatory responses, and suppression from specialized, regulatory T and B cells (1). Pattern recognition receptor (PRR) responses are broadly suppressed in the fetus in early gestation, but the mechanisms involved are unclear (2, 3). Specifically, these responses become activated in the early third trimester of gestation in humans, following a hierarchical emergence pattern, with cytosolic/intracellular PRR showing activity arising before extracellular PRR in neonatal monocytes (4–6). These developmental changes are coordinated, such that, at the term of gestation, neonatal myeloid cells are able to produce robust responses that prepare the newborn for de novo exposure to microorganisms (7, 8). Although these functional changes have been well described, the molecular mechanisms underlying the regulation of innate immune responses early on, during human development, remain poorly understood.

Cellular metabolism plays an important role in regulating innate immune responses in general and, specifically also, during early ontogeny (2, 9). During homeostasis, immune cells engage energetically efficient oxidative phosphorylation in the mitochondria for ATP production. Robust inflammatory responses following PRR stimulation require rapid energy production, with upregulation of biosynthesis pathways through cytoplasmic glucose metabolism; conversely, alternative activation is regulated through mitochondrial pathways (10). When oxygen supply becomes limited, significant changes in metabolic pathway activity occur, with increased reliance on glycolysis and remodeling of the mitochondrial network by altering mitochondrial dynamics (11, 12). In utero, oxygen saturation is physiologically low (13), which raises the possibility that distinct cell-intrinsic mechanisms may be required to adapt to the metabolic conditions of the fetal environment. While physiological hypoxia maintains (hematopoietic) stem cells in a quiescent state (14), pathological hypoxia can also suppress homeostatic monocyte functions (15). mTORC1 is a key signaling hub for maintaining hematopoietic stem cell quiescence and stemness through limitation of mitochondrial biogenesis (16). mTORC1 activity is reduced in neonatal monocytes and monocyte-derived macrophages (2, 9). Additionally, our previous data indicated a potential role for DDIT4L in this context, as its expression was found to be increased in neonatal monocytes in unbiased genome-wide gene expression experiments (2).

DDIT4L, a paralog of DDIT4, negatively regulates mTORC1 (17, 18), which, in turn, is a key regulator of downstream processes such as proliferation, protein translation, and cell-intrinsic metabolism (19). In mice, targeted deletion of the *Ddit4* gene reprograms bone marrow–derived macrophages toward a glycolytic phenotype and enhances LPS-induced IL-1β responses (20). In cardiomyocytes, diverse metabolic stress conditions, such as glucose or serum deprivation, cellular oxidative stress, or DNA damage, caused upregulation of DDIT4L (21). These data suggest that DDIT4L may play an important role in modulating cellular metabolic stress responses and innate immune activity during development.

Here, we tested whether DDIT4L can directly regulate innate immune responsiveness in human neonatal myeloid cells. Using a combination of molecular, functional, and metabolic studies, comparing primary preterm, term, and adult monocytes, we demonstrate a gestational age–dependent reduction in mitochondrial capacity in neonatal monocytes. Furthermore, we demonstrate that DDIT4L is selectively upregulated under metabolic stress conditions in neonatal monocytes. To provide more direct mechanistic insights into the potential role of DDIT4L in this context, we created genetically modified monocytic U937 cell clones. Biochemical, metabolic, and imaging studies using these cells confirmed that DDIT4L upregulation reduced mitochondrial mass, altered regulators of mitochondrial dynamics, and blunted LPS cytokine responses. Further analysis showed that *DDIT4L* is upregulated in lung macrophages from premature neonates during the neonatal period, with reduced expression of this gene in infants who develop bronchopulmonary dysplasia, suggesting a potential protective role against inflammatory-associated lung injury. Altogether, these data point toward a developmental role for DDIT4L in regulation of mitochondrial and innate immune activity in myeloid cells as well as adaption to metabolic stress and prevention of pathological inflammation during the fetal and early neonatal transition period.

## Results

### Oxidative metabolism and mitochondrial activity are reduced in neonatal monocytes.

**Figure 1:**
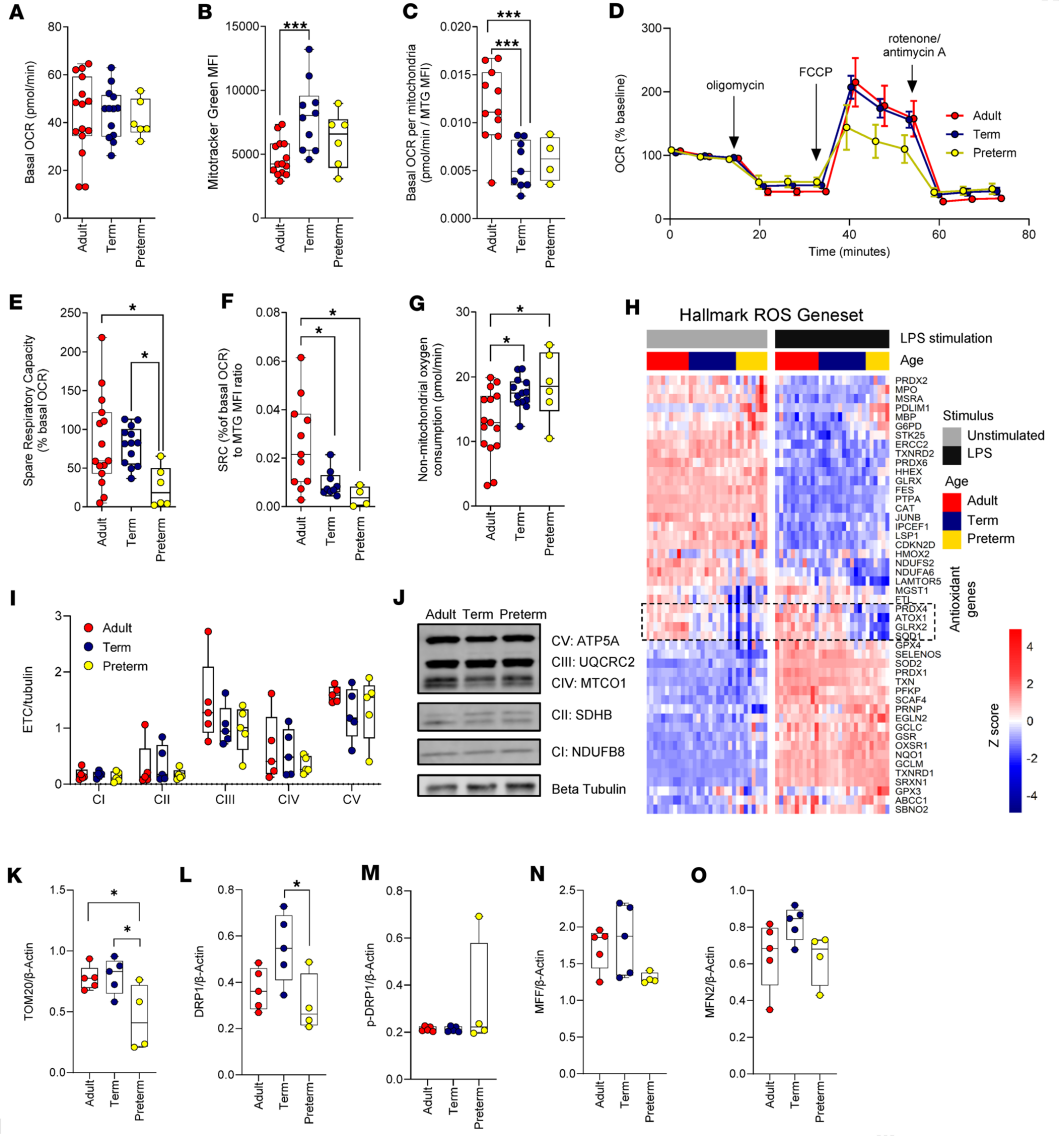
Reduced mitochondrial activity and capacity in neonatal monocytes. (**A**) Mitochondrial activity (basal oxygen consumption rate [OCR]), (**B**) mitochondrial mass (Mitotracker Green staining by flow cytometry; mean fluorescent intensity [MFI]), and (**C**) ratio of basal OCR to mitochondrial mass (when sufficient samples were available for paired analyses) in adult, term, and preterm monocytes. (**D**) Mitochondrial stress test (mean with 95% confidence interval and with *x* axis values nudged by 1 minute between age groups to enhance visibility), with (**E**) spare respiratory capacity (SRC) and (**F**) the ratio of SRC to mitochondrial mass (when sufficient samples were available for paired analyses). Data are from 15 adult, 13 term, and 6 preterm samples. (**G**) Nonmitochondrial oxygen consumption from same samples as in **A**, **D**, and **E**. (**H**) Expression of ROS genes (Hallmark pathway), using data from GEO accession GSE104510 (2). Data from 11 (LPS) to 12 (unstimulated) adults, 12 term and 6 (LPS) to 8 (unstimulated) preterm samples. (**I**) Electron transport chain proteins (by Western blot; data are from 5 adult, term, and preterm neonatal samples), including (**J**) a representative blot (of 5) of regulators and indicators of mitochondrial fission and fusion (**K**) TOM20, (**L**) DRP1, (**M**) DRP1^S616^ phosphorylation, (**N**) MFF, and (**O**) MFN2 in primary monocytes (see Supplemental Figure 2B for raw Western blot images). Data are from 5 adult, 5 term, and 4 preterm neonatal samples. For **A**–**G**, **I**, and **K**–**O**, data are represented as boxes (25th to 75th percentile) and whiskers (minimum to maximum), with medians indicated by solid lines. Groups were compared using 1-way ANOVA with Tukey’s multiple comparisons tests between age groups. For **I**, groups were compared using 2-way ANOVA, with nonsignificant age effect (*P* > 0.05). *P < 0.05, ***P < 0.001; only significant *P* values are shown.

Neonatal monocytes and monocyte-derived macrophages displayed reduced mTOR and glycolytic activity at lower gestational age (2, 9). At baseline, oxygen consumption rates (OCRs) were comparable in preterm, full-term neonatal, and adult monocytes (Figure 1A). However, when normalized to mitochondrial mass, as measured by MitoTracker Green, neonatal monocytes showed significantly less respiration (Figure 1, B and C). Additionally, spare respiratory capacity (SRC) was further reduced at lower gestational age, as shown by a profound reduction of respiration in response to carbonyl cyanide-p-trifluoromethoxyphenylhydrazone (FCCP, an ionophore that uncouples mitochondrial oxidative phosphorylation from ATP production), specifically in preterm neonatal monocytes, compared with term or adult monocytes (Figure 1, D and E). Normalization of SRC [calculated as (OCR after FCCP – basal OCR)/basal OCR × 100] to mitochondrial volume revealed that adult monocyte mitochondria were considerably more efficient than their neonatal counterparts, implying that higher mitochondrial mass may partly compensate for a reduced mitochondrial efficiency at term (Supplemental Figure 1; supplemental material available online with this article; https://doi.org/10.1172/jci.insight.172312DS1), but this efficiency was not seen earlier in gestation in preterm monocytes (Figure 1F). These data revealed a developmental gradient in mitochondrial respiratory capacity in myeloid cells.

Consistent with gestational age–dependent changes in mitochondrial activity in primary human neonatal monocytes, preterm neonatal monocytes also showed increased nonmitochondrial oxygen consumption (Figure 1G) and reduced expression of antioxidant genes, such as microsomal glutathione *S*-transferase 1 (*MGST1*), peroxiredoxin 4 (*PRDX4*), and superoxide dismutase (*SOD1*) (Figure 1H). These differences may indicate that preterm monocytes may be less resilient to hyperoxia. Furthermore, there were no differences in expression of mitochondrial electron transport chain complex proteins (Figure 1, I and J), supporting that the reduced mitochondrial reserve capacity in neonatal monocytes may be more related to altered mitochondrial morphology, as the former was previously shown to correlate with altered mitochondrial morphology in primary T cells (22). Indeed, when examining expression profiles of genes involved in mitochondrial fusion, gestational age–dependent developmental differences were observed, with preterm monocytes standing out, compared with term and adult monocytes (Supplemental Figure 2A).

The reduced mitochondrial activity and respiratory capacity in neonatal monocytes was also supported by significantly reduced levels of translocase of the outer mitochondrial membrane 20 (TOM20) in preterm monocytes, a proxy often used for mitochondrial mass (Figure 1K and Supplemental Figure 2B). Furthermore, total dynamin-related protein 1 (DRP1) was significantly reduced in preterm monocytes compared with term monocytes (Figure 1L), although we could not reproducibly detect the fission-associated phosphorylated form of DRP1^ser616^ (Figure 1M). Expression of the mitochondrial fission factor (MFF) was also reduced in preterm monocytes, and this was especially evident in the high-molecular-weight splice isoforms, although these differences were not significant, possibly due to the small number of sample replicates (Figure 1, M and N, and Supplemental Figure 2B). Expression of the mitochondrial fusion protein mitofusin-2 (MFN2) did not differ between the age groups (Figure 1O). Overall, our data could suggest that lower gestational age is associated with altered mitochondrial dynamics and reduced mitochondrial efficiency (22, 23). Yet, these data also support a higher propensity for ROS generation and oxidative stress in early gestation monocytes (24) in the context of reduced mitochondrial efficiency.

### Metabolic limitations reduce cytokine production and induce DDIT4L expression.

**Figure 2:**
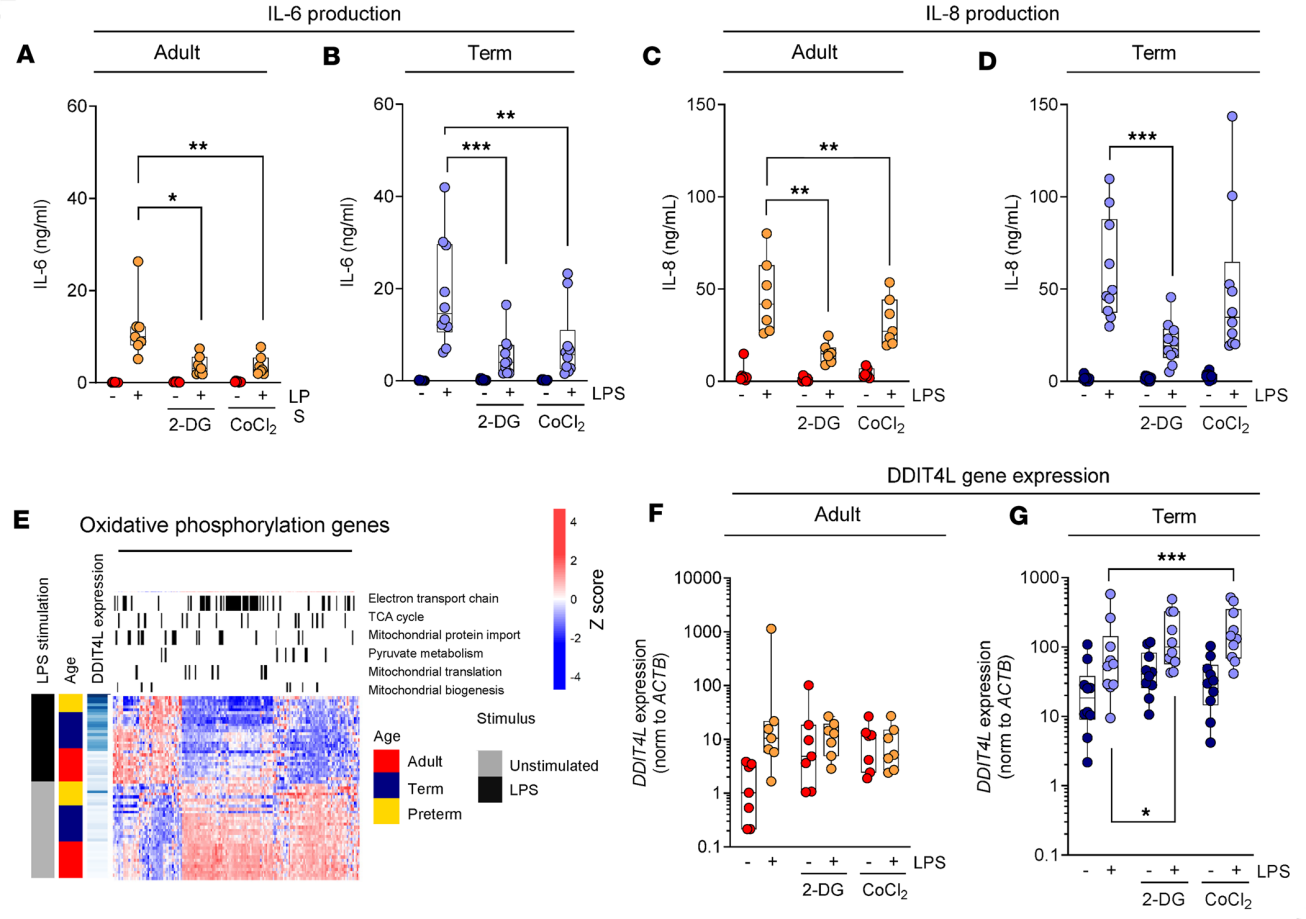
Metabolic stress is associated with increased DDIT4L expression and reduced cytokine responses in neonatal monocytes. (**A** and **B**) IL-6 and (**C** and **D**) IL-8 cytokine production by adult and term neonatal monocytes after stimulation with LPS (5 hours) in the presence of 2-deoxy-glucose (2-DG) or cobalt chloride (CoCl_2_). (**E**) Reduced expression of electron transport chain, tricarboxylic acid (TCA) cycle, mitochondrial protein import, pyruvate metabolism, mitochondrial translation, and mitochondrial biogenesis genes in adult, term, or preterm monocytes with or without LPS stimulation (5 hours), in relation to *DDIT4L* gene expression using data published in GEO accession GSE104510 (same as Figure 1H). The genes from the Hallmark pathway “Oxidative phosphorylation” with annotation using Reactome pathways (black line indicates gene is a member of that pathway) are shown. (**F** and **G**) *DDIT4L* gene expression (by qPCR, normalized to *ACTB*) from samples in **A**–**D**. Data are from 11 (LPS) to 12 (unstimulated) adults as well as 12 term neonatal and 6 (LPS) to 8 (unstimulated) preterm neonatal samples in **E** and from 7 adults and 10 term neonatal samples in **A**–**D**, **F**, and **G**. Data are represented as boxes (25th to 75 percentile) and whiskers (minimum to maximum), with medians indicated by solid lines. Groups were compared using 2-way ANOVA (on log-transformed data), yielding significant effect of metabolic stress conditions (2-DG or CoCl_2_ versus control) in the neonatal (*P* = 0.0297) but not in the adult (*P* = 0.3676) LPS-stimulated samples (*P < 0.05, **P < 0.01, ***P < 0.001).

Next, we sought to determine whether limiting metabolic capacity in monocytes was associated with altered innate immune function. To this end, (adult and neonatal) monocytes were stimulated with LPS in the presence of metabolic stress: exposure to CoCl_2_, an agent that induces chemical hypoxia, or 2-DG, a glucose analog that inhibits cellular glucose metabolism. Both significantly reduced production of IL-6 and IL-8 in adult and neonatal monocytes upon LPS stimulation (Figure 2, A–D). Previously, we had shown that the negative mTORC1 regulator DDIT4L is upregulated in neonatal monocytes, particularly in the preterm age group (2). As its paralog DDIT4 has been shown to limit mitochondrial activity, we hypothesized a potential link between DDIT4L expression and the metabolic phenotype in neonatal monocytes. Leveraging transcriptomic data, we observed that LPS-induced DDIT4L upregulation was associated with changes in the expression of oxidative phosphorylation genes in neonatal monocytes, particularly before 33 weeks of gestation (Figure 2E and Supplemental Tables 1 and 2). The upregulation of DDIT4L following LPS stimulation also negatively correlated with inflammation and interferon response genes in preterm monocytes (Supplemental Table 2). Interestingly, exposure to 2-DG and CoCl_2_ also similarly led to further upregulation of DDIT4L expression in neonatal, but not in adult, monocytes, suggesting an age-dependent developmental response to metabolic stress (Figure 2, F and G). The *DDIT4L* gene was also highly expressed in neonatal granulocytes, suggesting a conserved developmental function more broadly across other myeloid cells (Supplemental Figure 3). Altogether, these data linked DDIT4L to metabolic stress and impaired mitochondrial and immune activity.

### DDIT4L limits mitochondrial activity and inflammatory responses.

The aforementioned experiments linked DDIT4L to metabolic stress in neonatal myeloid cells. However, they did not distinguish whether the changes in mitochondrial dynamics and innate immune activity are a cause or a consequence of the upregulation of DDIT4L. To mechanistically address the effect of DDIT4L, we transduced U937 monocytic cells using a lentivirus vector in which expression of the *DDIT4L* coding sequence was regulated by a doxycycline-inducible promoter. To exclude substantial endogenous DDIT4L expression, wild-type U937 cells were compared with U937 cells in which the endogenous *DDIT4L* gene was knocked out using CRISPR/Cas9. In doing this, we detected no endogenous DDIT4L expression (Supplemental Figure 4). In parallel, we tested whether LPS-induced cytokine responses were mTOR dependent in another premonocytic cell line (THP-1 cells); however, in preliminary experiments we identified that the DDIT4L-targeted mTOR activity was dissociated from LPS-induced cytokine responses in THP-1 cells, as evidenced by a lack of suppression of IL-8 and TNF-α cytokine production when cells were also treated with rapamycin (Supplemental Figure 5). Consequently, U937 cells were used for further experiments, instead of THP-1 cells, as the experimental human cell model.

**Figure 3:**
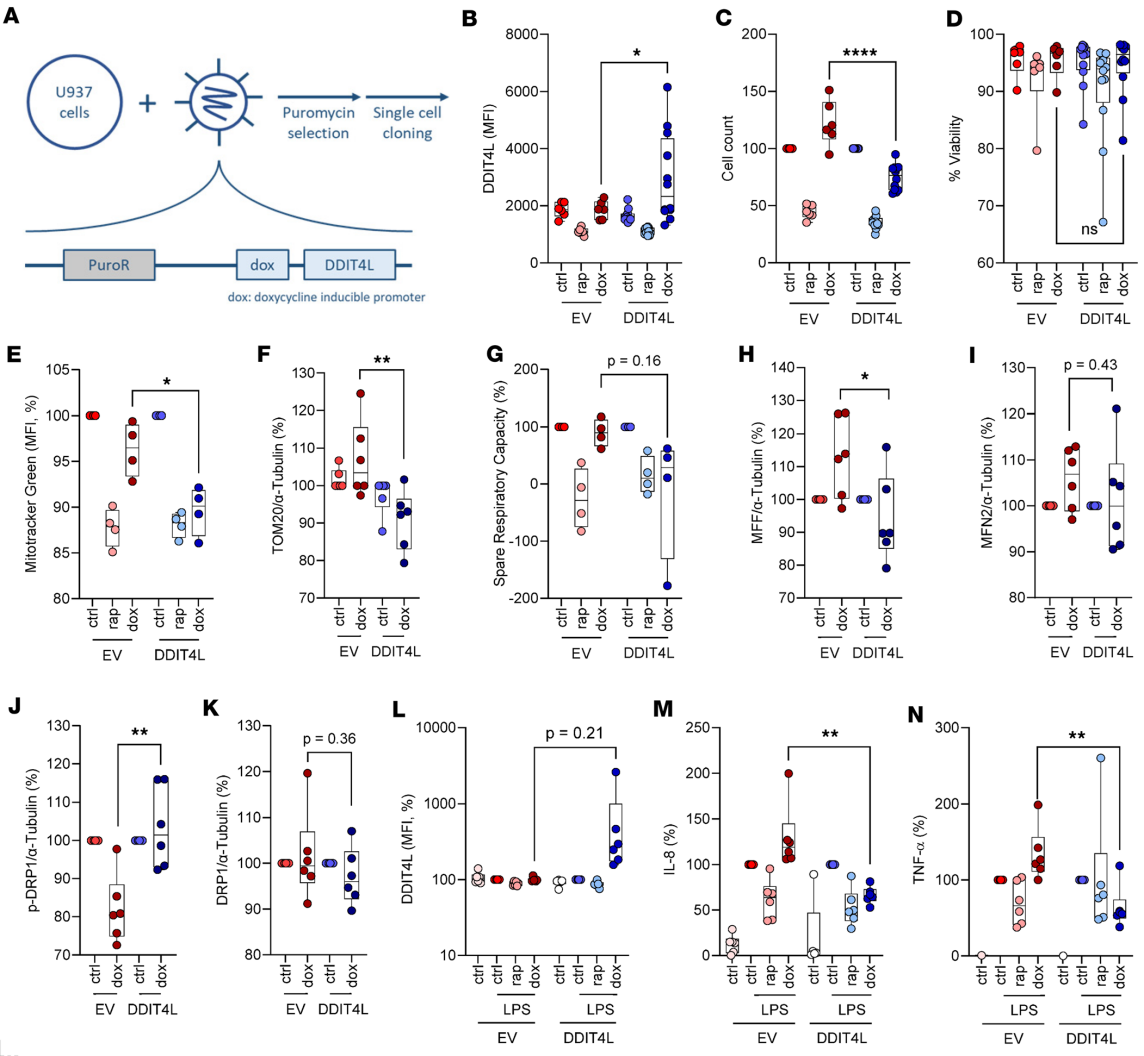
DDIT4L overexpression alters mitochondrial dynamics, suppresses mitochondrial activity, and inhibits cell proliferation and LPS cytokine responses in U937 cell clones. (**A**) Schematic representation of the generation of DDIT4L-overexpressing U937 clones. (**B**) DDIT4L protein expression, (**C**) cell count, (**D**) cell viability and (**E**) mitochondrial mass (by flow cytometry), and (**G**) spare respiratory capacity (by mitochondrial stress test on Seahorse analyzer) following culture of single-cell-sorted selected DDIT4L- or empty vector–transduced (EV-transduced) U937 cell clones for 72 hours in the presence of rapamycin (rap, 10 ng/mL), doxycycline (dox, 100 ng/mL), or a corresponding concentration of DMSO, as control (ctrl). Data are from 6 EV and 12 DDIT4L independent cell clones in **B**–**D**, 4 EV and 4 DDIT4L independent cell clones for **E** and **G**, and 6 EV and 6 DDIT4L independent cell clones for **F** and **H**–**N** (representative data from 1 of 3 experiments, using separate batches of the same clones). (**L**) DDIT4L expression (by flow cytometry), (**M**) IL-8, and (**N**) TNF-α production (by ELISA) in PMA-differentiated (48 hours) DDI4L- or EV-transduced U937 cell clones treated overnight with rap, dox, or control conditions followed by LPS stimulation for 5 hours. Protein expression of mitochondrial fission and fusion markers: (**F**) TOM20, (**H**) MFF, (**I**) MFN2, (**J**) DRP1 phosphorylatioin and (**K**) DRP1 (Supplemental Figure 8 for corresponding Western blots) in DDI4L- or EV-transduced U937 cell clones after overnight culture in the presence of dox or DMSO control conditions. Data are presented as boxes (25th to 75th percentile) and whiskers (minimum to maximum), with solid lines indicating the median, normalized to DMSO conditions. *P* values were calculated using 2-sided unpaired *t* tests for specific differences between EV-dox and DDIT4L-dox experimental conditions (Welch’s correction was applied to **B** and **L** due to unequal variances between the 2 comparison groups; data for DDIT4L expression in **L** was log transformed prior to statistical testing). Only relevant comparisons between EV-dox and DDIT4L-dox are show for simplicity. *P < 0.05, **P < 0.01, ***P < 0.001, ****P < 0.0001.

Following lentivirus transduction of DDIT4L in U937 cells, single cells were sorted, and clones were expanded and selected for expression of increased protein levels of DDIT4L upon doxycycline treatment (Figure 3, A and B). In parallel, we also generated U937 clones transduced with an identical vector construct that lacked the *DDIT4L* coding sequence as a control (empty vector [EV]). All experimental conditions were compared with cells treated with the mTOR inhibitor rapamycin (10 ng/mL) as a positive control. Notably, rapamycin, but not DDIT4L, treatment reduced U937 cell size by about 20% (measured by flow cytometry; Supplemental Figure 6). DDIT4L-transduced cell clones also showed reduced proliferation with prolonged doxycycline treatment over 72 hours, compared with EV clones (Figure 3C), without impacting the viability of the transduced cell clones (Figure 3D). We also observed modest effects of doxycycline treatment on cell size and proliferation in the EV clones, after 72 hours of treatment (Supplemental Figure 6 and Figure 1C). These experiments demonstrated the importance of comparing all conditions to corresponding EV control conditions to correctly interpret data and quantify potential effects of DDIT4L on cellular functions and metabolism.

**Figure 4:**
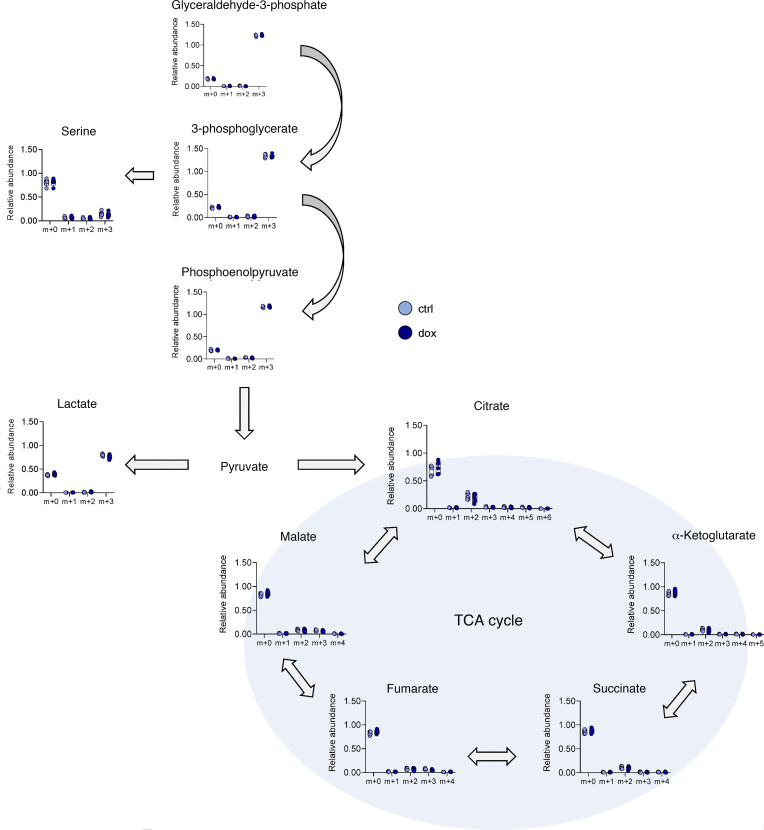
Effect of DDIT4L overexpression on central carbon metabolism. U937 cell clones transduced with DDIT4L were cultured overnight in the presence of doxycycline (dox, 100 ng/mL) or an equivalent amount of DMSO (control condition; ctrl) and 10 mM ^13^C-labeled glucose. Metabolites were detected on a gas chromatography mass spectrometry. Mass isotopologue distributions of selected metabolite ion fragments were quantified and corrected for natural isotope abundance using algorithms adapted from ref. 41. Data were averaged from 5 DDIT4L clones treated with doxycycline or an equivalent of DMSO overnight (12 hours, 1 experiment) and are presented as the relative abundance of each mass (m) isotope.

Using nonquantitative confocal microscopy imaging studies, we show that DDIT4L expression (after overnight treatment of cells with doxycycline) altered the mitochondria morphology to more elongated and filamentous shapes and that there was an overall lower mitochondrial volume, as shown by HSP60 staining (Supplemental Figure 7). Flow cytometry and Western blot experiments demonstrated that DDIT4L overexpression significantly reduced mitochondrial mass (as shown by reduced MitoTracker Green staining and TOM20 expression in Figure 3, E and F) and lowered mitochondrial respiratory capacity (Figure 3G); this was also consistent with observations in primary neonatal monocytes. Moreover, experiments showed that DDIT4L overexpression altered molecular regulators of mitochondrial dynamics, as evidenced by changes in the relative expression of fission/fusion proteins, namely reduced expression of the high-molecular-weight MFF isoforms and increased phosphorylation of DRP1 (Figure 3, H–K, and Supplemental Figure 8). Given the changes in mitochondrial content, we assessed glucose carbon assimilation in central carbon metabolism, with a focus on glycolysis and the mitochondrial tricarboxylic acid cycle. Functionally, DDIT4L overexpression significantly reduced LPS-induced IL-8 and TNF-α to levels similar to those of the U937 clones stimulated with LPS in the presence of rapamycin (Figure 3, L–N). These data experimentally confirmed a causal role for DDIT4L in shaping mitochondrial dynamics and activity and suppressing LPS-stimulated cytokine responses. Thus, the phenotype of DDIT4L-expressing U937 cell clones closely mirrored the phenotype detailed above in preterm neonatal monocytes. Despite lower mitochondrial content and indications of altered mitochondrial dynamics, previously shown to alter glucose allocation in T cells (22), we did not observe a marked effect of DDIT4L induction on glucose-derived carbon allocation by quantification of heavy-labeled glucose by gas chromatography mass spectrometry (GC-MS) (Figure 4). The latter experiments suggested that the relative glucose allocation to lactate, the tricarboxylic acid cycle, and serine biosynthesis were not altered by DDIT4L overexpression.

### DDIT4L expression during bronchopulmonary dysplasia in preterm infants.

**Figure 5:**
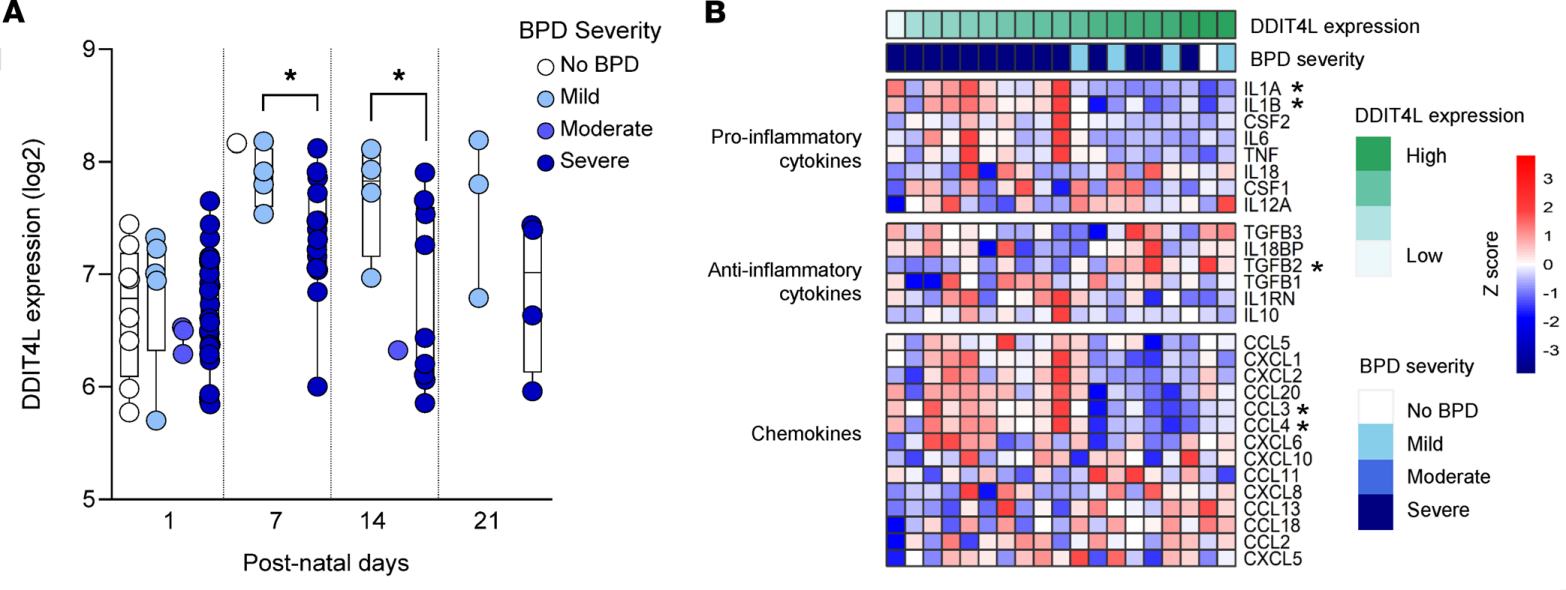
Low DDIT4L gene expression is linked to severe bronchopulmonary dysplasia in preterm neonates during the neonatal period. (**A**) DDIT4L expression in lung macrophages over a range of postnatal days, between neonates with no BPD or those with mild, moderate, or severe BPD. Data were obtained from GEO accession GSE149490 and included 4–25 infants per bronchopulmonary dysplasia [BPD] severity group, but the same number of samples were not necessarily available for all time points, as samples were collected only in infants that were endotracheally intubated for ventilation. Significant differences in *DDIT4L* gene expression are shown at postnatal day 7 and 14 (at the time when early BPD clinical signs usually develop; severity groups were compared at each postnatal day using a 2-sided unpaired *t* test with Welch’s correction for unequal variance; *P < 0.05). (**B**) Expression heatmap of cytokine and chemokine genes (same data set) on postnatal day 7 (1–14 infants per BPD severity group) showing decreased proinflammatory cytokine and chemokines and increased antiinflammatory cytokine gene expression with increasing *DDIT4L* gene expression (*statistically significant at FDR 5%). Additionally, see Supplemental Table 3 for pathways correlating with *DDIT4L* gene expression.

To address the in vivo relevance of these observations, we examined publicly available transcriptome data from macrophages obtained from lung aspirates of neonates born prematurely during the neonatal period. Intriguingly, DDIT4L expression in lung macrophages increased in the first 2 weeks of postnatal age in preterm neonates, consistent with a developmental role for DDIT4L, at this early life stage (Figure 5A). Moreover, *DDIT4L* gene expression in lung macrophages also appeared reduced in infants who developed more severe bronchopulmonary dysplasia (BPD), an inflammatory-associated form of chronic lung disease, compared with infants with mild or no BPD (Figure 5A). Consistent with a potential role for DDIT4L in limiting innate immune activation in myeloid cells, high *DDIT4L* gene expression correlated negatively with the expression of proinflammatory cytokine and chemokine genes, such as *IL1B* and *CCL3,* and positively with expression of antiinflammatory cytokine genes, such as *TGFB2*, on postnatal day 7 (Figure 5B). Additionally, *DDIT4L* gene expression was negatively correlated with the Hallmark pathways “TNF-α signaling via NF-κB” and “inflammatory response” (Supplemental Table 3). Together, these data possibly implicate DDIT4L in the regulation of potentially tissue-damaging inflammation mediated through myeloid cells in preterm infants during the early neonatal period.

## Discussion

**Figure 6:**
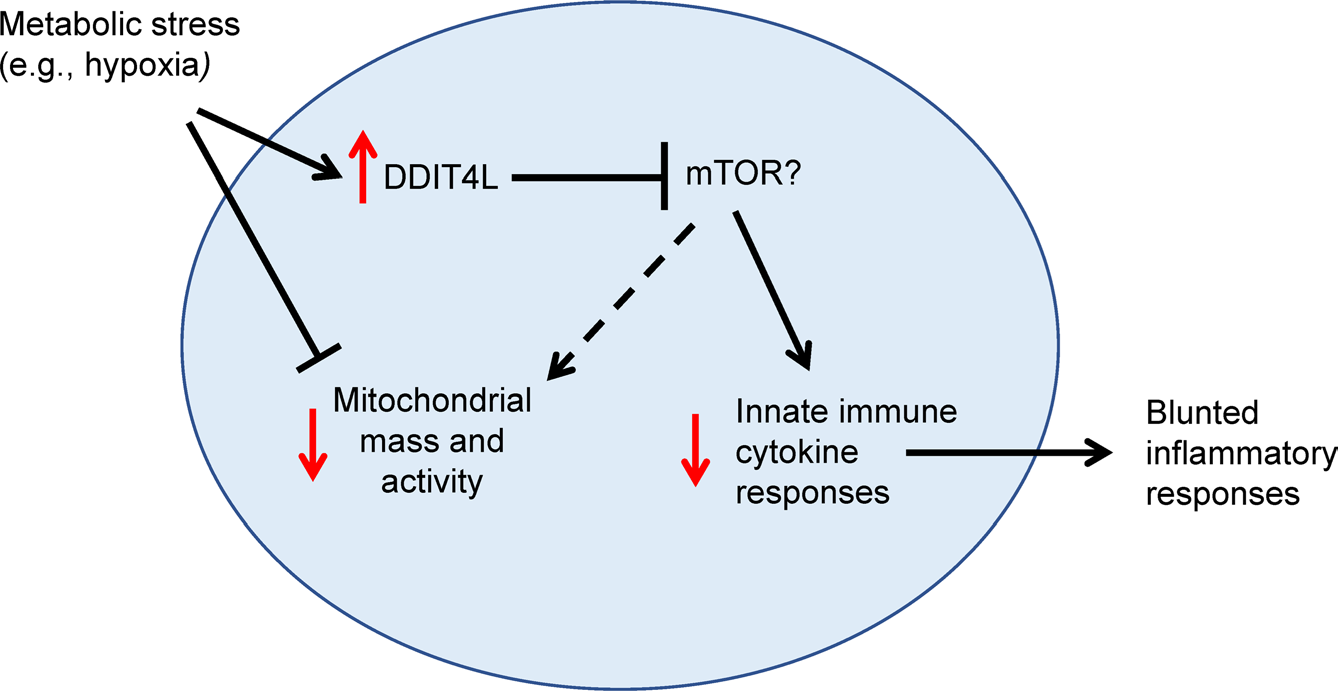
Model for DDIT4L regulation of innate immune responses in human myeloid cells during fetal development and the early neonatal period in preterm neonates. We hypothesize that the effect of DDIT4L upregulation on reducing mitochondrial mass and cellular metabolic activity may be particularly important in conditions of metabolic stress such as physiological hypoxia in utero.

The data presented here confirm a direct role for DDIT4L in regulating mitochondrial dynamics and innate immune activity in myeloid cells, providing the first evidence to our knowledge for a potential role during fetal and neonatal development. The study of human primary neonatal monocytes is extremely challenging for obvious ethical and practical reasons, especially early in gestation, due to the low blood volumes obtainable in small newborns. To overcome this challenge, we combined key descriptive studies in primary neonatal monocytes and granulocytes with more mechanistic experiments to determine the causal impact of DDIT4L overexpression using genetically engineered monocytic cell clones. Transcriptome and real-time metabolic activity studies showed profoundly reduced mitochondrial respiration capacity in neonatal monocytes with a phenotype more pronounced in earlier gestation. In U937 cells, DDIT4L overexpression altered mitochondrial dynamics and blunted LPS responses, mirroring observations in neonatal monocytes. These data support a model whereby DDIT4L may play an important role during the fetal and early transition neonatal period, suppressing innate immune activation and mitochondrial activity (Figure 6). Dampening mitochondrial activity may be essential to avoid energy-costly immune responses stress in the low-oxygen fetal environment and prevent oxidative stress upon the sudden exposure to ambient air at birth. In light of these findings, we speculate that DDIT4L helps prevent potentially harmful innate immune activation and the cellular consequence of mitochondrial energy failure in the physiological hypoxic fetal environment, while preparing the newborn for the neonatal transitions. To the best of our knowledge this is the first evidence implicating DDIT4L in human development and the first evidence directly demonstrating its direct role affecting mitochondrial dynamics and innate immune activity in myeloid cells.

DDIT4L expression can be induced in response to cellular stresses, such as DNA damage, hypoxia, hypoglycemia, serum starvation, or arsenite (25, 26). The paralogs DDIT4 and DDIT4L have been most extensively studied in skeletal muscle tissue (27), and DDIT4 has also received considerable attention relating to its role in cancer and diabetes (26). Through inhibition of mTOR, the paralog DDIT4 remodels cellular metabolism, increasing autophagy and mitochondrial activity while suppressing glycolytic energy pathways (25, 26, 28). Few studies have examined DDIT4L function in myeloid cells. Overexpression of DDIT4L (also known as REDD2 or RTP801L) in macrophages increased ROS production and cell death, particularly necrosis in response to oxidized LDL (18, 29). Homologs of DDIT4 and DDIT4L have been found in a variety of species, such as drosophila, cows, and mice, supporting an evolutionarily conserved role (17, 30). Another study showed upregulation of DDIT4L after LPS or zymosan stimulation of rainbow trout macrophages (31). However, the specific role of DDIT4L in human myeloid cells had been studied scarcely. Our results showing that DDIT4L overexpression blunts LPS-induced cytokine production are consistent with previous data showing that knockout of the paralog *Ddit4* increases IL-1β production and remodels cellular metabolism, including blunted mitochondrial activity, in murine bone marrow–derived macrophages (20, 32).

While we have attempted to overexpress or knockdown DDIT4L in primary monocytes (data not shown), major limitations in working with these cells — which terminally differentiate into macrophages quickly in vitro — and additional difficulties are rooted in obtaining sufficient primary monocytes, especially in preterm neonates, which precluded pursuing these experiments any further. Instead, we opted to address the direct effect of DDIT4L in U937 myeloid cell clones, a model previously used to overexpress DDIT4L (18). mTOR inhibition through DDIT4L overexpression only modestly reduced LPS-induced cytokines, which may be a limitation of this transformed cell line. Indeed, as cancer cells, U937 cells have a high metabolic rate (33), potentially limiting sensitivity to mTOR inhibition, and certainly differ metabolically from primary monocytes, at least in part, due to continuous proliferation. Thus, combining observations in primary cells with more mechanistic experiments in DDIT4L-inducible cells proved to be an effective approach to generate compelling data and insight into a potential developmental role of DDIT4L in human myeloid cells.

The precise mechanism of action of DDIT4L has scarcely been studied, but available data suggest that it limits mTORC1 activity, like its paralog DDIT4 (25, 34). Both proteins limit phosphorylation of mTORC1 and its downstream targets S6 and 4E-BP1 (17, 34). The exact mechanism through which DDIT4L acts on mTOR is unknown, but DDIT4L likely functions upstream of the TSC1/TSC2 complex (34). Potential modes of action have been reviewed for DDIT4 (25, 27, 28). Like DDIT4, DDIT4L may bind directly to members of the 14-3-3 scaffolding protein family, impeding their interaction with TSC1, thereby permitting the formation of a functional TSC1/TSC2 complex. However, direct protein-protein interaction between DDIT4L or DDIT4, and 14-3-3 has not been demonstrated and may appear unlikely according to 3D modeling, despite the presence of a 14-3-3 binding motif in DDIT4 (35). Another model suggests binding of DDIT4L to PP2A, leading to dephosphorylation and inhibition of AKT kinase. Yet it remains possible that DDIT4L may disrupt ER-mitochondrial-associated membranes by binding to GRP75, as described for DDIT4 (25, 26, 28). The data presented here are consistent with all of these proposed mechanisms. The observed alterations to regulators of mitochondrial dynamics in neonatal monocytes and in DDIT4L-overexpressing cells could be part of a cellular metabolic network that is engaged upon metabolic stress, such as the lack of oxygen and/or nutrient availability. The precise mechanism of how DDIT4L affects mitochondrial biology and whether the observed changes described above are downstream of mTORC1 inhibition remains unknown. Lack of nutrient availability, loss of mTORC1 signaling, or activation of AMPK as a result of a change in the metabolic environment is often associated with adaptations in mitochondrial morphology, often resulting in elongated and/or fused mitochondria with increased SRC. However, our data suggest that DDIT4L expression in neonatal monocytes or monocytic cell lines induces changes most often associated with mitochondria that have undergone fission, such as DRP1^ser616^ phosphorylation, and fits with the observed reduction in SRC. However, since increased DRP1^Ser616^ phosphorylation and reduced MFF are observed in DDIT4L-overexpressed U937 cell lines, it will be important to test how DRP1-MFF interactions are regulated and whether the altered high-molecular-weight MFF isoforms play a role in mitochondrial dynamics and result in altered monocyte function. Exactly how DDIT4L alters mitochondrial parameters and how this intersects with the existing literature on DDIT4L require further study. The cell model we have generated here may help address these mechanistic molecular questions. Given the complexity of cell-intrinsic metabolic regulation of immune cell function, and the need for large numbers of cells to uncover causal metabolic changes that impact cytokine secretion, the development of cell-line-based reductionist models is a key asset that will allow us to expand our mechanistic understanding of how DDIT4L affects monocyte metabolism and function.

The gene expression data in tracheal aspirates from preterm infants showed upregulation of DDIT4L in the early postnatal period. The observation that DDIT4L expression was reduced in infants who subsequently developed neonatal chronic lung disease further suggests an important biological role of DDIT4L in limiting the exaggerated inflammation during the neonatal transition period (36, 37). In addition to supporting the biological relevance of our observation, these findings may point toward worthwhile therapeutic interventions. In humans, lungs mature mostly in the latter stages of gestation and after birth. Consequently, infants born very prematurely necessitate extensive respiratory support to maintain them alive in their early days. Under these conditions, the risk of BPD is further exacerbated by poor antioxidant defenses (36, 38). In this context, modulating innate immune responses in preterm myeloid cells may help limit the inflammation induced upon an oxidative stress during this critical development window period (37, 39). Our data also raise the far more ambitious clinical prospect that drugs targeting the DDIT4L-mTOR axis may reduce inflammation and prevent BPD in these infants.

## Methods

### Sex as a biological variable.

Deidentified cord blood samples from both sexes were included indiscriminately. However, sex was not considered as a biological variable in the analysis owing to the lack of information provided for cord blood and in view of the limited sample size for experiments.

### Human participants.

Peripheral blood was collected from healthy adults. Cord blood was collected at the term of gestation (37–41 weeks of completed pregnancy; following a cesarean section delivery) or before 33 weeks of gestation (for preterm samples; following cesarean section or vaginal delivery) at the British Columbia Women’s Hospital. All blood samples were collected in sodium heparinized vacutainers and processed within 2 hours of collection.

### Human primary cell culture.

Peripheral or cord blood mononuclear cells were isolated by density gradient centrifugation. Briefly, blood was diluted 1:1 with PBS (or 1:2 for cord blood) and layered on top of 30 mL Lymphoprep (Stemcell, 07861). After centrifugation for 20 minutes at 1,000 g without brake, mononuclear cell layer was extracted and washed twice with PBS before immediate usage of cells for experiments or cryogenic storage. Where indicated, monocytes were extracted using the CD14^+^ purification kit II (Stemcell Technologies; 17858). After removal of the mononuclear cell layer, the remaining red blood cells and granulocyte were diluted 1:1 with PBS, and red blood cells were sedimented with 3% Dextran (MilliporeSigma, 31392) for 30 minutes at room temperature. Granulocytes were collected and residual red blood cells were lysed with 0.2% NaCl for 30 seconds. Mononuclear cells, monocytes, or granulocytes were cultured in round-bottom 96-well plates in RPMI with 10% human AB serum (Gibco, 11875-093), 1% sodium pyruvate, and 1% penicillin/streptomycin.

### Cell lines.

U937 cells were cultured in RPMI supplemented with 10% FBS (Thermo Fisher Scientific, 26140079), 1% sodium pyruvate, and 1% penicillin/streptomycin and generally seeded at 0.1 million cells per mL for passages every 2–3 days. For lentivirus-transduced cells, 1 μg/mL puromycin (MilliporeSigma, P8833-10MG) was added to the culture media. DDIT4L expression was induced by addition of 100 ng/mL doxycycline (Peprotech, 2431450) for indicated times. For generation of lentiviral particles, HEK293T cells were grown in IMDM with 10% FBS, 1% sodium pyruvate, and 1% penicillin/streptomycin. Cell cultures were regularly confirmed to be free of mycoplasma contamination. Cell line authentication was not performed.

### CRISPR/Cas9 generation of DDIT4L-knockout cells.

We generated *DDIT4L* gene–knockout cells by transfecting wild-type U937 cells with pX458 (Addgene 48138) containing DDIT4L guide RNA (5′-ATTTCAGAATTGCTGGACTG-3′ or 5′-CAAACTGCCAGTTGCAACCA-3′) and Cas9-2A-GFP. Following nucleofection (Lonza 4D Nucleofector, 1 million cells in 100 μL SF solution, program code FF100), cells were rested overnight before single-cell sorting of GFP^+^7-AAD^–^ cells. Recovered clones were screened for knockout by PCR and Sanger sequencing. Expression of DDIT4L in clones with *DDIT4L* coding frame sequences diverging from the reference genome were assessed for DDIT4L expression by flow cytometry using a PE-conjugated anti-DDIT4L antibody (Thermo Fisher Scientific, MA5-28628, clone DDIT-03, DDIT4L-PE used at 1:25 dilution).

### Lentiviral transduction of U937 cell clones.

To generate the *DDIT4L*-encoding lentiviral vector, the luciferase coding sequence was removed from pCW57.1-Luciferase (Addgene 99283) and replaced with the *DDIT4L* open reading frame or ligated to generate pCW57.1-*DDIT4L* and pCW57.1-EV, respectively. Correct insertion of intact open reading frame was confirmed by sequencing. Lentiviral particles were generated using calcium chloride transfection of HEK293T cells as described previously (40). Supernatant was harvested 2 days after transfection and concentrated by ultracentrifugation.

U937 cells were transduced by a *DDIT4L*-encoding or corresponding EV (same lentivirus construct without the *DDIT4L* coding sequence) lentivirus vector by spinoculation (60 minutes at 800*g* at 32°C) in the presence of 8 μg/mL polybrene (MilliporeSigma, TR-1003-G). Medium was changed the next morning, and puromycin was added at 2 μg/mL on day 2 after transduction. After 2 weeks of antibiotic selection, DDIT4L- or EV-transduced cells were single-cell sorted. Clones with varying levels of DDIT4L expression after induction with doxycycline (5 hours, 100 ng/mL) were selected.

### Flow cytometry.

Surface staining for CD14 (Biolegend, 325628, clone HCD14, CD14-BV421 used at 1:100 dilution) was performed in 50 μL PBS for 15 minutes at room temperature in the dark. For intracellular staining for DDIT4L (Thermo Fisher Scientific, MA5-28628, clone DDIT-03, DDIT4L-PE used at 1:25 dilution), the FOXP3 transcription buffer kit was used (Thermo Fisher Scientific, 00-5523-00). Cells were fixed in 100 μL 1X fix buffer (Thermo Fisher Scientific, 00-5523-00) for 15 minutes at room temperature in the dark. After washing with 100 μL 1X permeabilization buffer (Thermo Fisher Scientific, 00-5523-00), antibodies were added in 25 μL or 50 μL permeabilization buffer for 15 minutes staining at room temperature in the dark. Subsequently, cells were washed twice and stored in 2% PFA in PBS at 4°C until acquisition on a BD LSR II or Fortessa X-20 at the British Columbia Children’s Hospital Research Institute Core Technologies and Services. Flow cytometry data were analyzed using FlowJo version 10. Viability stains (7-AAD or fixable viability dye in eF506 or eF780) were included in all assays. Analyses were performed on single, viable cells.

### Metabolic assays.

200,000 CD14^+^ cells per well in triplicate were analyzed on a Seahorse XFe96 analyzer (Agilent Technologies) by performing the mitochondrial stress test according to manufacturer’s instructions. For baseline mitochondrial mass assessment, mononuclear cells were plated in cRPMI in round-bottom 96-well plates and incubated with 100 nM MitoTracker Green (Thermo Fisher Scientific, M7514) for 30 minutes at 37°C. Cells were washed 3 times prior to surface staining with CD14-BV421 (Biolegend, 325628) as described in the flow cytometry section above. After staining, cells were kept on ice and acquired within 2 hours. For metabolic assessment of the cell line clones, U937 cells were cultured overnight in the presence of 100 ng/mL doxycycline, 10 ng/mL rapamycin, or DMSO. Next, cells were stained with TMRE (Cayman, 21426, final concentration 50 nM), and MitoTracker Green (final concentration 100 nM) for 15 minutes at 37°C, 5% CO_2_. Cells were washed twice, 7-AAD was added, and cells were acquired immediately.

### GC-MS.

U937 cell clones transduced with DDIT4L were cultured overnight in the presence of doxycycline (100 ng/mL) or DMSO and 10 mM universally ^13^C-labeled glucose. Cells were then collected, treated with cold Methanol (80% Methanol/20% H_2_O), and extracts were dried using a speedvac. Dried extracts were incubated with MOX for 1 hour at 40°C followed by a 1-hour incubation with tBDMS. Samples were then run on GC-MS (Agilent Technologies) by the Analytical Core for Metabolomics and Nutrition at the British Columbia Children’s Hospital Research Institute. Metabolites were identified using retention times and *m/z* in Supplemental Table 4. Mass isotopologue distributions of selected metabolite ion fragments were quantified and corrected for natural isotope abundance using algorithms adapted from ref. 41. The code is available on https://github.com/Sethjparker/IntegrateNetCDF_WithCorrect/commit/4b742505b8575b2438b1a697b70480754ad93b4f (commit ID: 4b74250) (accessed on November 21, 2023) under a Massachusetts Institute of Technology license.

### DDIT4L gene expression.

Monocytes were stimulated with 10 ng/mL LPS (Invivogen, tlrl-eblps) for 5 hours in the presence of 250 μM CoCl_2_ or 10 mM 2-DG (Fisher Scientific, ac111980050). After stimulation, cells were harvested into TRIzol (Thermo Fisher Scientific, 15596-026) for RNA extraction. RNA was transcribed into cDNA using the AB cDNA synthesis kit (Thermo Fisher Scientific, 4368814). qPCR was performed in triplicates using Sybr Green (Thermo Fisher Scientific, 4368706) on a ViiA 7 instrument (Applied Biosystems) for *DDIT4L* (primers, 5′-GTTTGAGCAGCAAGAACCCG-3′ and 5′-AGTCAAAATCACTTAGCAGGCTC-3′), *ACTB* (primers, 5′-CCTTGCACATGCCGGAG-3′ and 5′-ACAGAGCCTCGCCTTTG-3′), *IL6* (primers, 5′-CCCAGGAGAAGATTCCAAAGAT-3′ and 5′-ACTCTTGTTACATGTCTCCTTTCT-3′), and *RPLP0* (primers, 5′-TGTCTGCTCCCACAATGAAAC-3′ and 5′-TCGTCTTTAAACCCTGCGTG-3′). Data are represented as 2^–ΔΔCt^.

### Western blot.

For Western blot experiments, monocytes were washed with PBS and lysed in RIPA buffer supplemented with sodium orthovanadate, phenylmethylsulfonyl fluoride (PMSF), and protease inhibitor (Santa Cruz, sc-24948). U937 cells were washed with PBS and lysed in cell lysis buffer (Cell Signaling; catalog 9803) supplemented with PMSF. Following 3 rapid freeze-thaw cycles (dry ice to 37°C water bath), lysates were spun at 21,000*g* for 15 minutes. Cleared lysates were transferred to new prechilled tubes and stored at –80°C. Lysates were combined with Laemmli buffer and incubated for 5 minutes at 40°C before loading on 4%–20% Tris-Glycine gels (Thermo Fisher Scientific, XP04202BOX). PVDF membranes were blocked with 5% BSA for 2 hours before incubation with OXPHOS antibody cocktail (Abcam, ab110413, used at 1:1,000 dilution) and β-tubulin (Abcam, ab6046, used at 1:1,000 dilution) overnight. Incubation with secondary antibodies was performed for 1 hour (IRDye 680RD goat anti-mouse IgG and IRDye 800CW donkey anti-rabbit IgG, Licor Biosciences, 925-68070 and 925-32213, both at 1:10,000). Blots were imaged on a LI-COR Odyssey 9120 and analyzed using Image Studio version 5.2. For the assessment of proteins involved in mitochondrial dynamics in Western blots, the following antibodies (all from Cell Signaling Technology unless otherwise noted) were used: TOM20 (42406T, 1:2,000), total DRP1 (8570T, 1:2,000), p-DRP1 S616 (4494S, 1:1,000), MFF (84580T, 1:2,000), MFN2 (11925T, 1:2,000), β-actin (4970T, 1:2,000), and α-tubulin (MilliporeSigma, T9026, 1:10,000). All primary antibody incubations were followed by incubation with secondary HRP-conjugated anti-rabbit (Invitrogen, 31460) or anti-mouse antibodies (Invitrogen, 31430) in 5% milk and 0.1% Tween-20 in TBS (1:15,000) and visualized on radiosensitive film (Diamed) using chemiluminescent substrate (SuperSignal 763 West Pico or Femto, Thermo Fisher Scientific).

### Cell proliferation.

Cells were seeded at 100,000 cells in 5 mL R10 plus DMSO (vehicle control), 10 ng/mL rapamycin, or 100 ng/mL doxycycline and cultured for 72 hours in 6-well plates. Cells were transferred to 15 mL tubes and centrifuged (400*g* for 4 minutes) to remove media. Next, cells were divided between the following experiments: DDIT4L expression and cell count/viability measurement. For DDIT4L expression, cells were stained as described in *Flow cytometry*. For the cell count quantification, a known amount of liquid counting beads (BD Biosciences, 335925) was added to quantify cell numbers. Viability (7-AAD staining), cell counts, and cell volume (FSC-A) were assessed concurrently in the same sample preparation.

### Cytokine production.

200,000 U937 cells were differentiated with 100 ng/mL PMA (MilliporeSigma, P1585-1MG) in 1 mL R10 in 24-well plates for 48 hours. Then, medium was replaced with 200 μL fresh R10 and stimulated with 100 ng/mL LPS for 5 hours. The night before LPS stimulation, DMSO, rapamycin, or doxycycline were added where indicated. Reagent concentration was maintained when changing the media for LPS stimulation. Following 5-hour LPS stimulation, supernatants and cells were harvested. Cells were trypsinized and stained for DDIT4L as described in *Flow cytometry*. Supernatants were stored at –80°C until cytokine quantification was performed by ELISA (Thermo Fisher Scientific, 88-8086-88 and 88734686) following manufacturer’s protocol.

### Confocal microscopy.

U937 cell clones transduced with DDIT4L were cultured overnight in the presence of doxycycline (100 ng/mL) or an equivalent amount of DMSO. Cells were transferred to cytoslides precoated with poly-D-lysine (MilliporeSigma, P6407) using a cytospin set to low acceleration, spinning for 4 minutes at 400 rpm. Cells were fixed with 4% paraformaldehyde for 10 minutes. After washing with PBS, cells were permeabilized in 0.1% digitonin containing 2% bovine serum albumin/PBS for 45 minutes. Then, cells were incubated with an antibody against HSP60 (1:250, Cell Signaling Technology, 12165S) to stain the mitochondrial matrix for 45 minutes. After washing with PBS, cells were incubated with Alexa Fluor 488–conjugated anti-rabbit secondary antibody (1:400, Cell Signaling Technology, 4412S) for 45 minutes in the dark. ProLong Diamond Antifade mounting media with DAPI (Thermo Fisher Scientific, P36966) was used for nuclear counterstains. Images were acquired using a SP5II Laser Scanning Confocal microscope (Leica), and representative 2-channel images were generated with Imaris imaging software (Oxford Instruments).

### Bioinformatic analyses.

The data presented include publicly available Gene Expression Omnibus (GEO) data sets GSE104510 and GSE149490. For GSE149490, samples were collected from tracheal aspirates obtained in premature neonates that were intubated, on the first day of life and on a weekly basis, only if the neonates remained intubated. Most infants remained intubated on days 14 and 21, while few to no samples from the “no BPD” group were available at later time points. Heatmaps were generated using the pheatmap package in R studio version 1.1.463 using R version 3.6.3. For assessment of genes coexpressed with DDIT4L, Spearman’s correlation was performed between DDIT4L and all other probes in the LPS-stimulated preterm samples (for GSE104510) or in the unstimulated day 7 samples (for GSE149490). The Spearman’s rho value was used as input for a preranked gene set enrichment analysis using GSEA software version 4.0.3 from the Broad Institute.

### Statistics.

Data were analyzed using unpaired 2-sided, 2-tailed *t* tests to compare specific 2-group combinations (e.g., EV-doxycycline versus *DDIT4L*-doxycycline), 1-way ANOVA with multiple comparison tests between each pair in experiments with 1 experimental group (e.g., to determine the effect of age), or 2-way ANOVA for experiments with multiple conditions (e.g., metabolic stress conditions and age). In each case, variances were assumed to be equal unless otherwise indicated. For data that were displayed on a log scale (e.g., DDIT4L protein expression), data were log transformed prior to testing. *P* < 0.05 was considered statistically significant. Data are represented as boxes (25th to 75th quartiles) and whiskers (minimum to maximum), with medians indicated by solid lines. Data were analyzed using GraphPad Prism. The statistical tests used in each case were indicated in the figure legends and include Tukey’s multiple comparisons tests and Welch’s correction in addition to tests listed above.

### Study approval.

This study was approved by the University of British Columbia Children’s and Women’s Research Ethics Board (H07-02681) in accordance with the declaration of Helsinki. Informed consent was obtained from adult participants and most parents of neonatal participants, except for a few cases where deidentified preterm cord blood samples were used following a waiver of consent when parents were unavailable and not reachable to provide consent, according to privacy procedures approved by our ethics research board.

### Data availability.

The accession numbers for the data sets are listed in the Supplemental Tables 1–3, the legends for Supplemental Figure 2, and Figures 1, 2, and 5 and include GEO GSE104510 and GSE149490. Any additional information required to reanalyze the data reported in this paper are available from PML upon request. Requests for further information or resources and reagents should be directed to and will be fulfilled by PML or RIKG.

## Author contributions

Study design was conceptualized by CM, PML, and RIKG, with methodological input from PCO, CJL, and AAS. CM, CC, JHO, EA, CRP, ASA, MAP, RDS, AM, MVP, RYX, FR, AEP, and LG performed experiments. Data were analyzed by CM, CC, JHO, CRP, ASA, MAP, and AM. Funding was acquired by PML and RIKG. CM, RIKG, and PML wrote the manuscript with input from all authors.

## Supplementary Material

Supplemental data

Unedited blot and gel images

Supplemental tables 1-4

Supporting data values

## Version 1 02/06/2024

In-Press Preview

## Version 2 03/08/2024

Electronic publication
