# Supplementary material for: DDIT4L regulates mitochondrial and innate immune activities in early life
Source: JCI Insight. 2024 Feb 6;9(5):e172312. doi: 10.1172/jci.insight.172312 (PMC11143921; doi:10.1172/jci.insight.172312)
Supplement: Supplemental data [file jciinsight-9-172312-s018.pdf]

Supplemental Figures

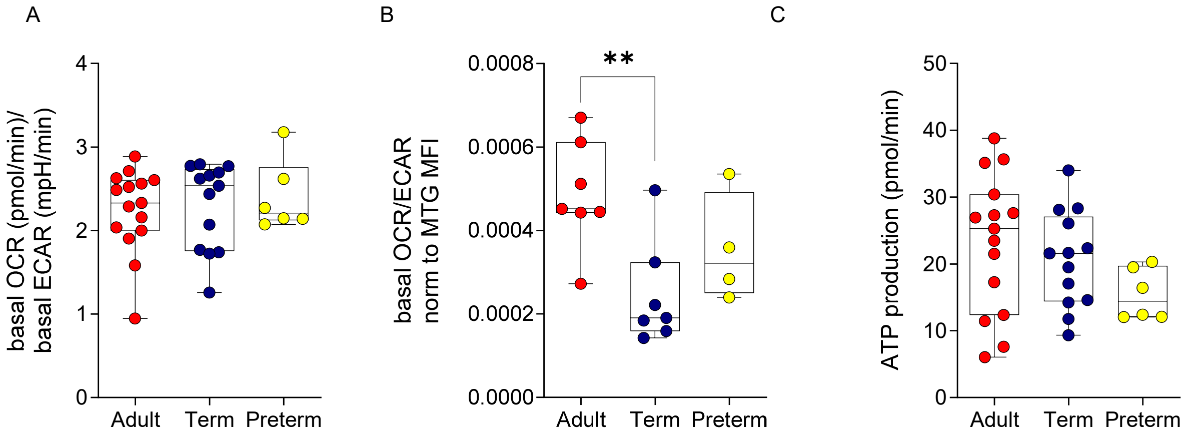

Supplemental Figure 1: Functional metabolic mitochondrial studies in primary monocytes. (**A**) Ratio of basal oxygen consumption rate (OCR) to Extracellular Acidification Rate (ECAR) in monocytes from adult, term and preterm. (**B**) Basal OCR/ECAR divided by MitoTracker Green (MTG) Mean Fluorescence Intensity (MFI) for individuals in which matched samples were available. (**C**) Mitochondrial ATP production (last rate measurement before oligomycin injection) – (Minimum rate measurement after oligomycin injection) from the same samples. Data was analyzed using one-way ANOVA followed by Tukey's multiple comparisons test. Data are from 15 adult, 13 term and 6 preterm subjects for A, C and from 11 adult, 9 term and 4 preterm paired subjects (for ratios) for B. Data are presented as boxes (25th to 75th percentile) and whiskers (min to max) with solid line indicating the median. Only significantly different comparisons are indicated (**p<0.01).

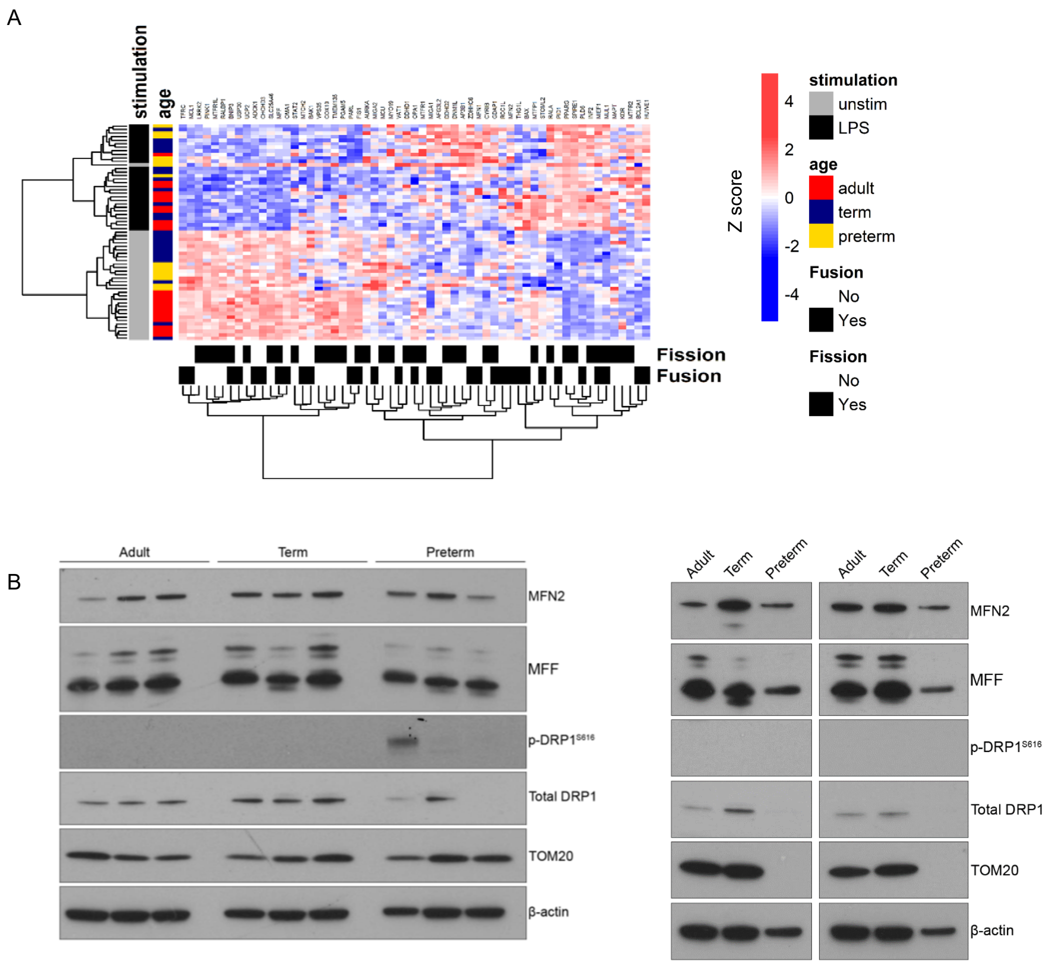

Supplemental Figure 2: Expression of mitochondrial fission and fusion protein markers in primary monocytes. (**A**) Differential expression of mitochondrial genes (black bars indicate fission and fusion group belonging, based on Gene Ontology Pathways), ± stimulation (5 h) from 11 (LPS) to 12 (unstimulated) adults, 12 term and 6 (LPS) to 8 (unstimulated) preterm samples, based on data from (Kan B et al., Nat Comm 2018) obtained from the Gene Expression Omnibus database under accession #GSE104510. (**B**) Raw Western blots corresponding to Figures 1K-O (5 individuals per age group). Note that the preterm samples in the second blot represent technical duplicates and were averaged for quantification.

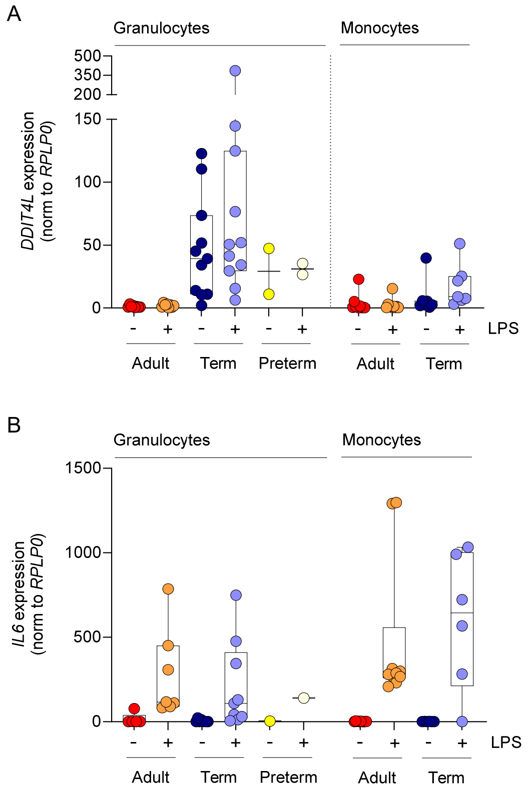

Supplemental Figure 3: Expression of DDIT4L in human granulocytes. *DDIT4L* (**A**) and *IL6* (**B**) gene expression normalized to *RPLP0* (by qPCR), in granulocytes and monocytes after a 5-hour stimulation with LPS (10 ng/mL) or no stimulation. Data are from 8 adults, 11 term and 2 preterm subjects combined from two separate experiments, represented as boxes (25th to 75th percentile) and whiskers (min to max) with solid line indicating the median.

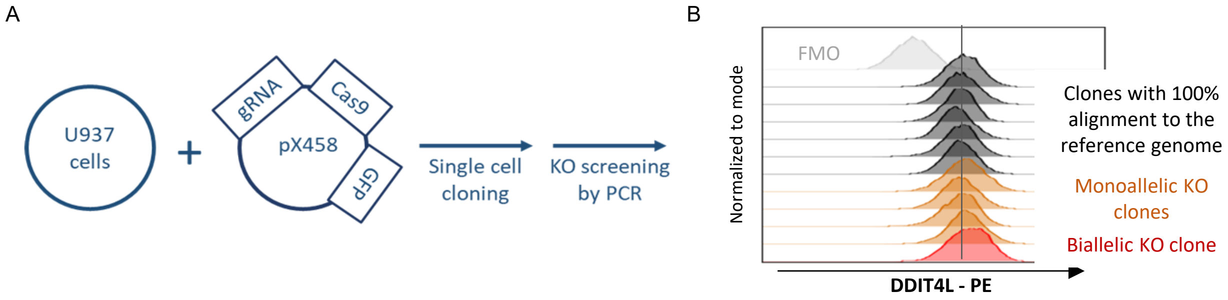

Supplemental Figure 4: Knock-out (KO) of DDIT4L using CRISPR, demonstrating that U937 cells express negligible endogenous levels of DDIT4L protein. (**A**) Strategy for generating DDIT4L KO clones and (**B**) histograms showing levels of DDIT4L protein expression between wild-type (black), partial (orange) and complete (red) KO (by flow cytometry staining of U937 cell clones using a PE-conjugated DDIT4L antibody). KO: Knock-out; GFP: green-fluorescence protein coding sequence. Px458 refers to plasmid sequence. GRNA: guide RNA.

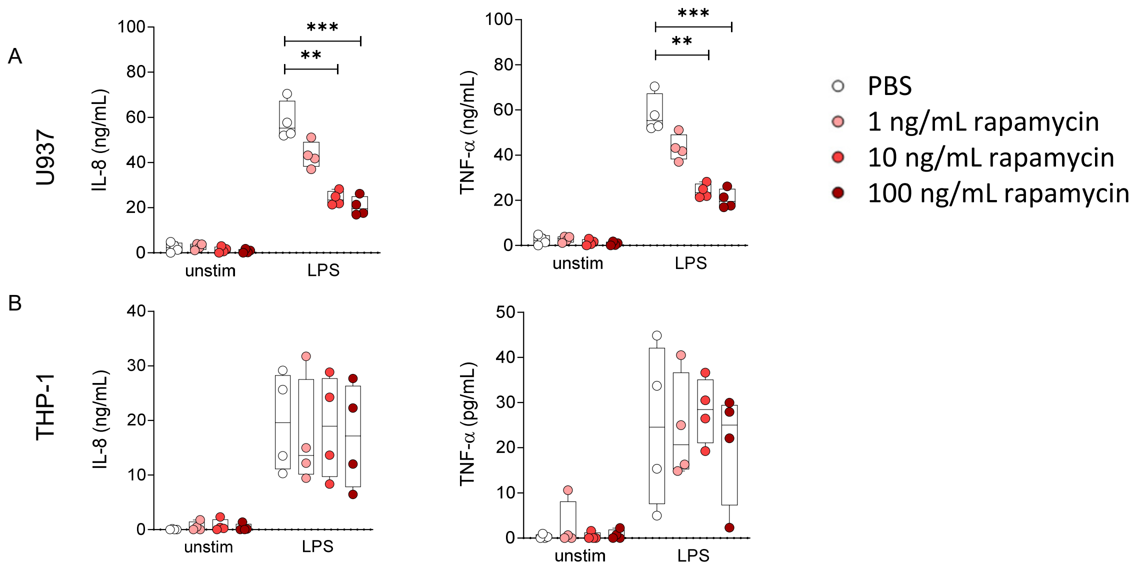

Supplemental Figure 5: Effect of rapamycin treatment on cytokine production in U937 and THP-1 cells. (**A**) U937 cells or (**B**) THP-1 cells were differentiated with PMA and stimulated with LPS (10 ng/mL) in the presence of PBS (control) or increasing doses of rapamycin. IL-8 and TNF-α were quantified by ELISA. Data represented as boxes (25th to 75th percentile) and whiskers (min to max) with median indicated by solid line. Data obtained from 4 different batches of PMA-differentiated U937 and THP-1 cells (4 independent experiments). LPS + rapamycin was compared to LPS alone by 2-sided paired t test. Only significant differences are shown (*$p<0.05$, **$p<0.01$, ***$p<0.001$).

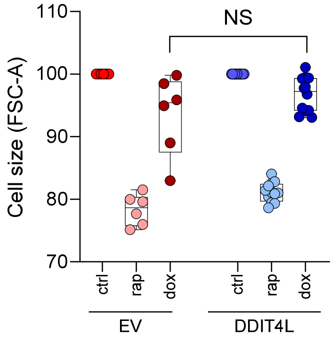

Supplemental Figure 6: Effect of rapamycin and DDIT4L overexpression on U937 cell size. U937 cell clones transduced with the lentiviral "empty vector" (EV) or the DDIT4L construct were cultured for 72 h in the presence of rapamycin (rap, 10 ng/mL), doxycycline (dox, 100 ng/mL) or an equivalent concentration of DMSO (ctrl). Cell volume was quantified by flow cytometry (FSC-A). Data are represented as boxes (25th to 75th percentile) and whiskers (min to max) with solid line indicating the median. Data are from 6 EV and 12 DDIT4L independent cell clones (data representative from 3 independent experiments using different batches of clones) and normalized to DMSO conditions. No significant (NS) difference was observed between EV dox and DDIT4L dox conditions ($p = 0.28$, by unpaired 2-sided t tests using Welch's correction for unequal variance).

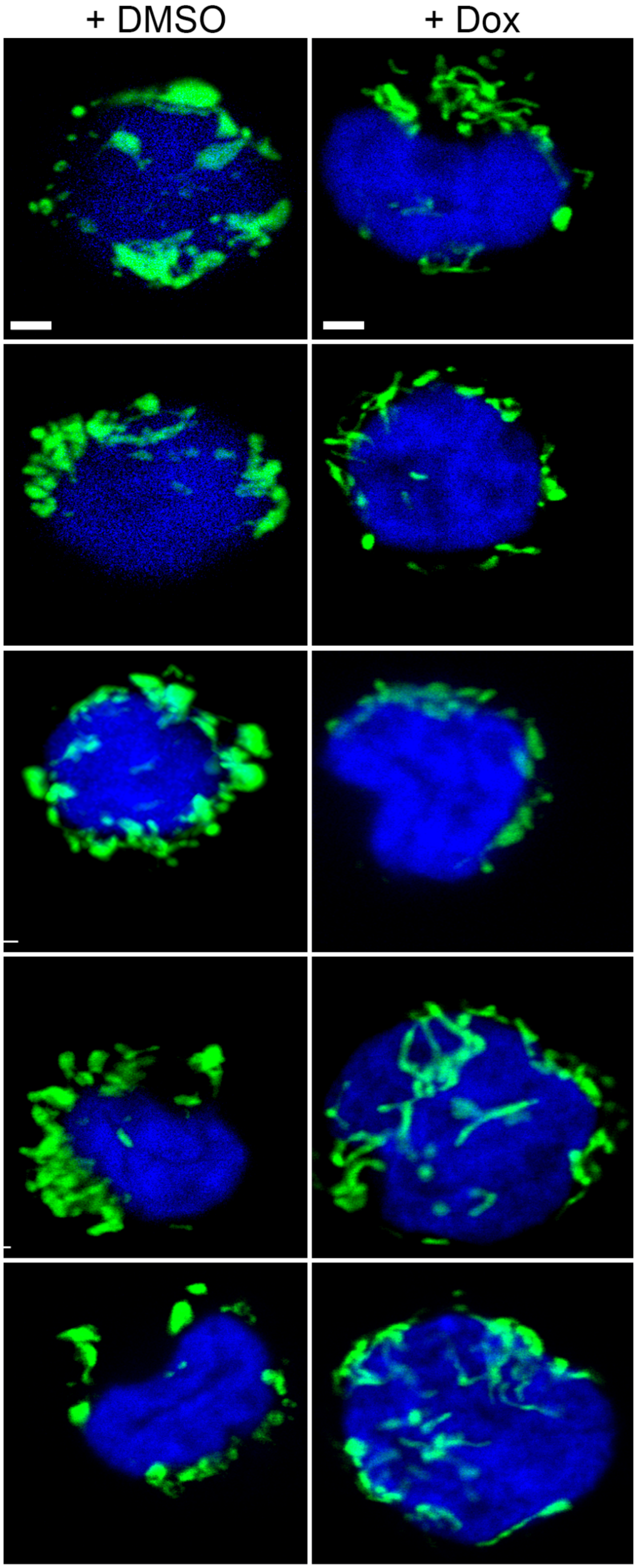

Supplemental Figure 7: Effect of DDIT4L overexpression on mitochondrial morphology. Representative confocal images of U937 cell clones transduced with DDIT4L after incubation overnight in the presence of doxycycline (dox, 100 ng/mL) or an equivalent amount of DMSO. 50,000 cells were spun onto poly-D-lysine coated cytoslides and fixed with 4% paraformaldehyde. Mitochondria were stained with Hsp60 antibody, followed by AF488 antibody and DAPI was used for nuclei staining. The scale bar is 2 µm. Images were acquired on a confocal microscope. Representative images from 5 DDIT4L U937 cell clones for doxycycline-induced or DMSO-treated cells stimulated in pairs (single experiment).

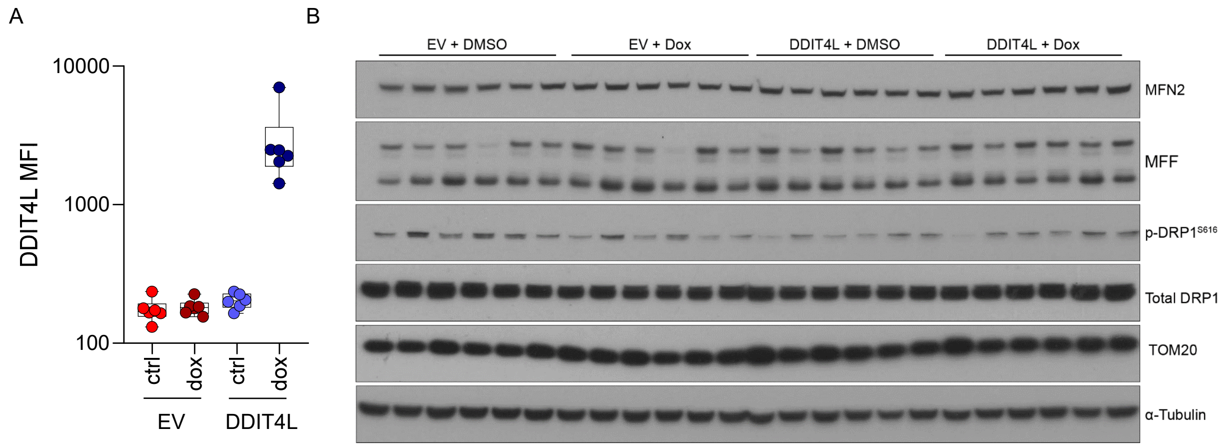

Supplemental Figure 8: Effect of DDIT4L overexpression on mitochondrial phenotype. (**A**) DDIT4L expression in U937 cell clones transduced with the DDIT4L gene or a corresponding “empty vector” (EV) vector after overnight culture in the presence of doxycycline (dox, 100 ng/mL) or an equivalent amount of DMSO (ctrl), used for Western blot experiments, with data represented as boxes (25th to 75th percentile) and whiskers (min to max) and solid line indicating the median. Data are from 6 EV and 6 DDIT4L independent cell clones (single experiment). (**B**) Corresponding raw Western blots to quantify mitochondrial fission and fusion protein markers.
