# Supplementary material for: DDIT4L regulates mitochondrial and innate immune activities in early life
Source: JCI Insight. 2024 Feb 6;9(5):e172312. doi: 10.1172/jci.insight.172312 (PMC11143921; doi:10.1172/jci.insight.172312)
Supplement: Unedited blot and gel images [file jciinsight-9-172312-s019.pptx]

## Slide 1
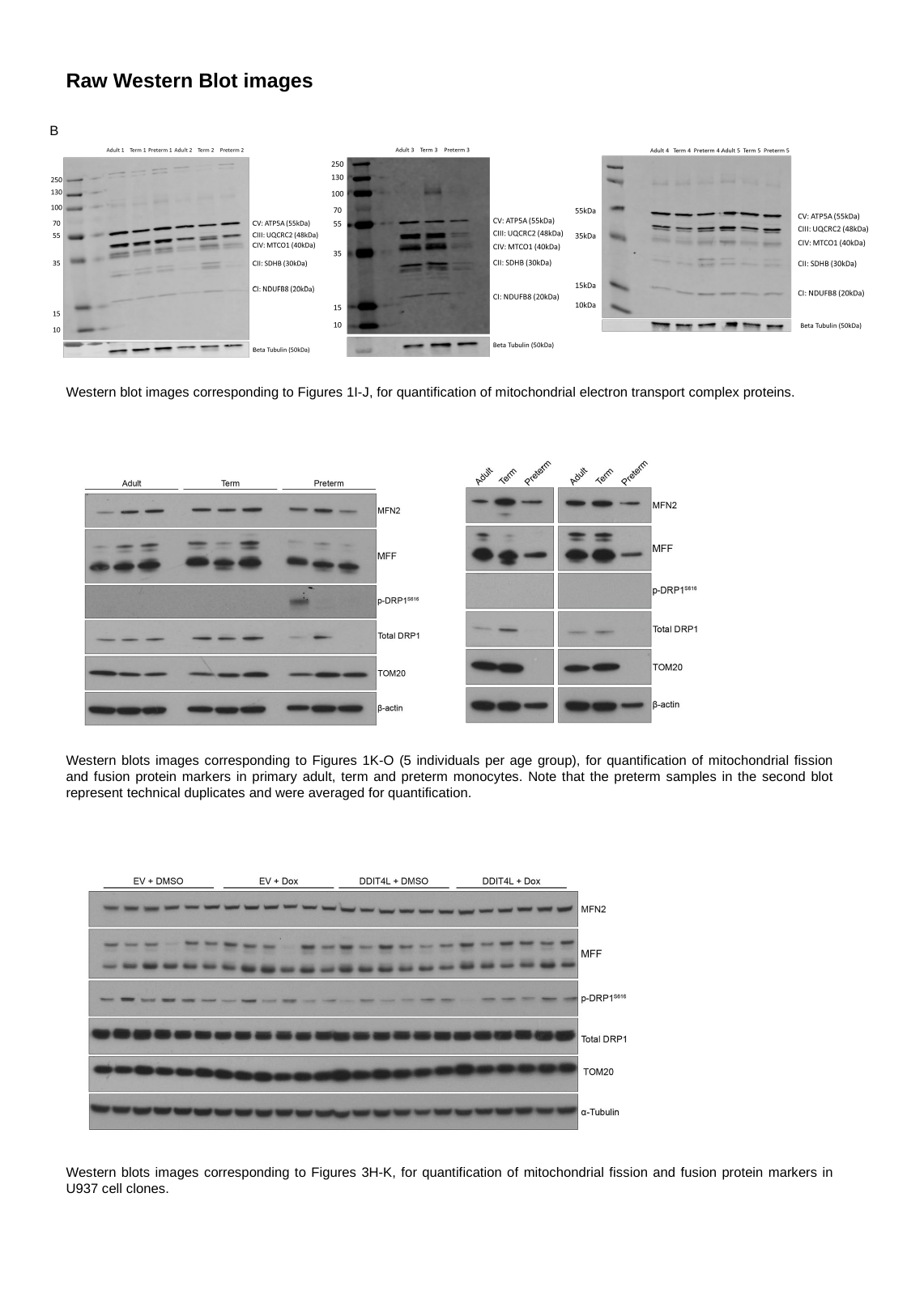

Raw Western Blot images
B
Western blot images corresponding to Figures 1I-J, for quantification of mitochondrial electron transport complex proteins.
Western blots images corresponding to Figures 1K-O (5 individuals per age group), for quantification of mitochondrial fission and fusion protein markers in primary adult, term and preterm monocytes. Note that the preterm samples in the second blot represent technical duplicates and were averaged for quantification.
Western blots images corresponding to Figures 3H-K, for quantification of mitochondrial fission and fusion protein markers in U937 cell clones.
